# Clinical Lecture at the College of Physicians and Surgeons, New York

**Published:** 1881-03

**Authors:** A. Jacobi


CHICAGO MEDICAL

JOURNAL & EXAMINER.

Vol. XLII.—MARCH, 1881.—No. 3.

### Original Lectures.

ARTICLE I.

Clinical Lecture Delivered December 8, 1880, at the College of Physicians and Surgeons, New York. By Dr. A. Jacobi. (Reported for the Chicago Medical Journal and Examiner.)

*Gentlemen* :—Here is a little girl whose shoulder was dislocated some time last week. She was brought to this clinic at that time and the luxation was reduced, but the arm cannot yet be moved without pain, and the shoulder is still swelled. This is evidently not precisely the condition which we should expect to find one week after the reduction of a simple dislocation. As I press the head of the humerus into the glenoid cavity, I feel a crackling sensation by which I know that there is some synovitis present. Now, what shall we do for that? The arm must have rest of course, but is there nothing else to be done? Counter irritation, yes; but what agent will you employ? Some one says, the tincture of iodine. In an older person that might do, but in children the skin is very delicate, and I think that the tincture of iodine would irritate too much. There is an officinal ointment of iodide of potassium, but I believe it to be nearly inert. I do not think that the drug could be discovered in the urine even after this preparation has been used for days, therefore I do not recommend it. But the iodide of potassium (1) may be dissolved in glycerine (2 parts) and rubbed into the skin. In that way it is effective; or iodine may be used dissolved in oleic acid, but there is an objection to that preparation, owing to the fact that the oleic acid sometimes cauterizes. What I frequently use is iodoform mixed with collodion, one to two parts of iodoform to twenty of collodion. You may cover the affected part with this preparation two or three times a day, or you may use the iodoform as an ointment. The chief drawback to the use of iodoform is its intolerable odor. The addition of a few drops of the aromatic oil of thyme or bergamot will render that less apparent. This child has, however, another ailment which you all doubtless recognize—whooping cough. It has only been manifest one week, the mother says, but in addition to that statement, she says that the child has had a cough for some weeks previous. Pertussis has a prodromal stage of about three weeks, during which it is impossible to diagnose it with certainty, but where the cough gets worse instead of better, and that in spite of good treatment, proper care, and rest at home, especially if there be whooping cough in the neighborhood, you may suspect whooping cough is present, and not an ordinary bronchitis. The whoop appears during the third week of the attack and is due to a spasm of the laryngeal muscles. Pharmacy has been exhausted in search of remedies for the disease, and almost everything has been given for the relief of the little patient, particularly the narcotics and sedatives. Chloral hydrate, since its introduction by Leibreich, has been used a good deal, but I use it only in single doses each night, giving seven or eight grains to a child a year and a half old. You may add fifteen grains of the bromide of potassium or sodium. Of the bromide of ammonium rather less, however. It has seemed to me that the sedative effect of the bromide of ammonium is less than that of either of the other salts. The attacks of coughing and laryngeal spasm are more frequent at night time than during the day. After making use of nearly all the drugs which have been recommended for pertussis the drug which I have found most successful is the one which I used many years ago, and that is belladonna. It always fails when the practitioner does not know how to use it. It must be given to do good, either in sufficient doses or not at all. To give drop doses of the tincture to this child would be useless. The drug must be given in doses sufficient to flush the cheeks, say four drops three times a day. If you find that within fifteen minutes after taking the dose the cheeks become flushed and remain so for half an hour afterward, the dose is sufficient. After three or four doses, you may have to increase the dose. Do so, drop by drop, as it is possible that within a fortnight you may be compelled to give three times the amount with which you started. You may never see dilatation of the pupils, dry throat or tongue, or very rarely, as it is not common for children to be so affected. This is about the only treatment which I have ever found effective. In the first week I expect no improvement, but in the second week I do, and at the end of the month I expect the cough to have ceased. This case appears to be a severe one because the child bleeds at the nose, which shows that there is some obstruction to the circulation in the upper part of the body. It is necessary for you to be on your guard whenever, in these cases, you have hæmorrhage occurring from mucous membranes, for you are likely to have also cerebral or spinal hæmorrhages, and these are more likely to happen according as the child is younger.

Case II. Norman N., five months old. "What is the matter with this child?" "He cries all the time." "Is there nothing else that you notice wrong about him?" "No, that is all." Now the baby cries very loud, as you see, and that very fact would exclude pneumonia or pleurisy as a cause of the child's suffering, for suffer he undoubtedly does, else he would not cry. The pain which causes him to cry must evidently be situated in such a part of the body as not to be increased by the movements of breathing. Were it in the chest—as every movement of the chest-wall would increase it—the child would not cry lustily as this child does, but feebly and with a short cry. The pain then must be situated in the joints or in the abdominal cavity. We can exclude earache, a very common cause of the incessant crying of children, because the cry *is* incessant always, which it is not in this case; besides, the child has had these fretful spells for some time, ever since she weaned him, which was some while ago, and if earache had been the cause of the child's suffering, it would have terminated in suppuration before now. The pains from which the child suffers are intestinal, and due to improper feeding, especially in relation to the interval between the meals. At first, the mother says, she had an insufficient supply of milk for the child, so that in the beginning the baby was starved. Then, after weaning him, she fed him on condensed milk. At present the bones of the head are soft. They may have softened after having once been normal, which is probably the case here. The mother says the child's bowels move only once or twice a day, which, in a child of this age, amounts to constipation. She also says in regard to the color of the passages that they are yellow, not white, but it never does to trust the statements of mothers implicitly, and I shall ask to see one of the passages. It is as I have said. The mother was in error when she said the passages were yellow. What I show you in this napkin is whitish, clay-colored, certainly not yellow. So the mother, knowing that children's passages are yellow, ordinarily when asked about her own child, says that his passages are yellow too. What, then, is the cause of these clay-colored stools? There is either something too much in the food, as fat or casein, or the liver secretes too little bile. I think that the child's food contains too much fat and casein both, and it appears to me that here is the root of the evil, the cause of the fretful disposition of this baby. The child has been fed on condensed milk diluted with water. Now, condensed milk contains an enormous amount of sugar, besides fat and casein. The normal passages contain eleven to sixteen per cent. of fat undigested, which proves that if mother's milk contained less fat it would still be as nourishing as now. The white appearances in this passage, are fat and coagulated casein. It would require the microscope to determine which preponderates. In cow's milk there is more fat than in mother's milk, and condensed milk of course contains fat and sugar in excess. Now, when there is so much fat in the passages, the fat and casein act as irritants in the intestinal canal, and, setting up a fermentation, give rise to flatulency, which of course in turn is the cause of much uneasiness and pain. There is the solution of the problem, the reason why this child crys so lustily and kicks so much. What is to be done to relieve him? If the cause of the trouble is an excess of fat, of course the first thing is to reduce the amount of fat which he is taking. Some one suggests that he should take as an antacid, lime water. Now, lime water contains only one part of lime to eight hundred parts of water, and consequently is not able to neutralize much acid, certainly not as much as is contained in the digestive tract of the child. It does good to be sure, but not in that way, but in a different direction. Lime water will aid in the digestion of casein, but it is slightly astringent and therefore ought not to be given in this case where constipation already exists. The use of condensed milk should be discontinued and fresh cow's milk should be substituted, with the addition of some oatmeal water, in this case because of the tendency to constipation. Take the common Irish meal and boil it in a sufficient quantity of water so that you have a transparent mucilaginous liquid. Strain it, and, adding a little salt and sugar, dilute an equal quantity of boiled milk with it and let the child take this preparation in place of the condensed milk. For the first few days, an occasional hot poultice to the bowels, or hot injections of fennel, or chamomile infusions, or catnip tea freshly made, will relieve the intestinal pains. Small doses of Dover's powder one-third to one-fourth of a grain, once or twice a day, will have a good effect. But of course the main thing which is necessary to restore this child to a condition of health is a complete and radical change in the diet, for as long as it continues to be fed on materials which it cannot assimilate, it will suffer from disturbances of digestion, and as a matter of course cannot thrive. (A week afterward the baby was presented with marked improvement.)

